# Co-Expression with Membrane CMTM6/4 on Tumor Epithelium Enhances the Prediction Value of PD-L1 on Anti-PD-1/L1 Therapeutic Efficacy in Gastric Adenocarcinoma

**DOI:** 10.3390/cancers13205175

**Published:** 2021-10-15

**Authors:** Ziqi Wang, Zhi Peng, Qiyao Liu, Zixia Guo, Merey Menatola, Jing Su, Ting Li, Qing Ge, Pingzhang Wang, Lin Shen, Rong Jin

**Affiliations:** 1 Key Laboratory of Carcinogenesis and Translational Research (Ministry of Education), Department of Gastrointestinal Oncology, Peking University Cancer Hospital & Institute, Beijing 100142, China; wzq2016@bjmu.edu.cn (Z.W.); zhipeng@bjmu.edu.cn (Z.P.); 2 NHC Key Laboratory of Medical Immunology, Department of Immunology, School of Basic Medical Sciences, Peking University, Beijing 100191, China; 1811110020@pku.edu.cn (Q.L.); 1410305104@pku.edu.cn (Z.G.); mereymee@bjmu.edu.cn (M.M.); liting@bjmu.edu.cn (T.L.); qingge@bjmu.edu.cn (Q.G.); wangpzh@bjmu.edu.cn (P.W.); 3 Department of Pathology, School of Basic Medical Sciences, Peking University, Beijing 100091, China; sujin@bjmu.edu.cn; 4 National Key Laboratory of Human Factors Engineering, China Astronauts Research and Training Center, Beijing 100094, China

**Keywords:** gastric adenocarcinoma, CMTM6, CMTM4, PD-L1, immunotherapy

## Abstract

**Simple Summary:**

Immunotherapeutic efficacy is low even in PD-L1 positive patients with advanced gastric adenocarcinoma. Based on the results of 6-color multiplex immunofluorescence staining of the gastric tumor tissues in tissue array and 48-case pre-immunotherapy patients, a better prognostic value was found in the membrane co-expression of CMTM6/4 and PD-L1 in tumor epithelial cells than PD-L1 alone. The membrane co-expression of CMTM6/4 and PD-L1 can be used as a valuable tool for precision pre-immunotherapy patient screening in gastric adenocarcinoma.

**Abstract:**

Anti-PD-1/L1 immunotherapy has been intensively used in heavily treated population with advanced gastric adenocarcinoma. However, the immunotherapeutic efficacy is low even in PD-L1 positive patients. We aimed to establish a new strategy based on the co-expression of CMTM6/4 and PD-L1 for patient stratification before immunotherapy. By analyzing the data obtained from TCGA and single-cell RNA sequencing at the mRNA level, and 6-color multiplex immunofluorescence staining of tumor tissues in tissue array and 48-case pre-immunotherapy patients at the protein level, we found that CMTM6/4 and PD-L1 co-expressed in both epithelial and mesenchymal regions of gastric adenocarcinoma. The tumor tissues had higher levels of CMTM6/4 expression than their adjacent ones. A positive correlation was found between the expression of CMTM6/4 and the expression of PD-L1 in tumor epithelium. Epithelial co-expression of CMTM6/4 and PD-L1 in gastric tumor region was associated with shorter overall survival but better short-term response to anti-PD-1/L1 immunotherapy. Thus, we developed a predictive model and three pathological patterns based on the membrane co-expression of CMTM6/4 and PD-L1 in tumor epithelial cells for pre-immunotherapy patient screening in gastric adenocarcinoma.

## 1. Introduction

Anti-PD-1/L1 immunotherapy has been used as a later-line therapy or first-line therapy in combination with chemotherapy for gastric cancer (GC), but with mixed therapeutic results. The CheckMate 032, CheckMate 649, ATTRACTION-2, ATTRACTION-4, KEYNOTE-012, KEYNOTE-059, and KEYNOTE-158 studies demonstrated anti-tumor activity and clinical benefit [1,2,3] while both KEYNOTE-061 and JAVELIN Gastric 300 studies failed to show the superiority of immunotherapy over chemotherapy [4,5]. PD-L1 expression is one of the primary biomarkers in predictive stratification of patients receiving anti-PD-1/L1 therapy [1,6,7]. However, its predictive value in highly dynamic and heterogeneous GC remains in question. PD-L1 expression was reported in 25–65% of GC patients by histochemical staining [8], while the high PD-L1 positivity did not correlate well with the response rate of anti-PD-1/L1 therapy in several clinical trials [2,5,9,10]. As the surface expression level of PD-L1 is regulated by multiple factors, investigating the molecules that promote the expression and/or stability of PD-L1 is thus important for the optimization of patient selection.

We previously identified nine members of CKLF-like MARVEL transmembrane domain-containing family (CMTM) (CKLF, CMTM1-8) in human cells. These molecules play critical roles in regulating the trafficking of transmembrane and secretory proteins [11,12]. Among them, CMTM6 and CMTM4 have been found to positively regulate the surface expression of PD-L1. CMTM6 interacts with PD-L1 in the plasma membrane and prevents PD-L1 from ubiquitin-mediated degradation. CMTM4 is closely related to CMTM6 and regulates PD-L1 expression in a similar way in head and neck squamous cell carcinoma and a CMTM6-depleted lung cancer cell line [13,14,15]. These data suggest that CMTM6/4 could be new biomarkers for patients that undergo anti-PD-1/L1 immunotherapy. High expression of CMTM6 has been found to correlate with poor prognosis in several types of tumor [16,17,18,19]. Its predictive value, however, remains controversial in GC. For example, high transcriptional levels of CMTM6/4 were associated with better survival in patients with GC [20], while high protein level of CMTM6 was associated with poor prognosis. Worse overall survival (OS) was further observed in patients with high expression of both CMTM6 and PD-L1 [21].

As CMTM6/4 regulates PD-L1 at the protein level, we investigated the prognostic value of the three molecules by multiplex immunofluorescence staining in gastric cancer tissue array. We also examined the predictive values of these molecules in response to anti-PD-1/L1 therapy in patients with GC. We found that the co-expression of CMTM6/4 and PD-L1 in gastric tumor epithelial cells significantly correlated with poor prognosis and may function as a pivotal predictor of response in GC patients receiving anti-PD-1/L1 immunotherapy. Therefore, a predictive model and three pathological patterns were developed for pathologists to evaluate the patients before immunotherapy.

## 2. Materials and Methods

### 2.1. Reagents and Antibodies

Primary antibodies to human cell markers for multiplex immunofluorescence staining are listed as follows: Anti-CMTM6 (HPA026980, Sigma-Aldrich. Shanghai, China), anti-pan-cytokeratin (ZM-0069, ZSGB-BIO, Beijing, China), anti-PD-1 (ZM-0381, ZSGB-BIO, Beijing, China), anti-PD-L1 (ZA-0629, clone SP142, ZSGB-BIO, Beijing, China). Anti-CMTM4 was produced by the Department of Immunology, Peking University Health Science Center.

### 2.2. Bioinformatics Analysis

Datasets of stomach cancer (STAD) from The Cancer Genome Atlas (TCGA) were downloaded from UCSC Xena web browser (https://xenabrowser.net/ (accessed on 28 October 2020)). Gene expression datasets of human cancer cell lines were directly downloaded from CCLE (https://portals.broadinstitute.org/ccle (accessed on 28 October 2020)) [22]. Single-cell datasets of raw UMI count matrices were derived from a recent study [23]. We utilized functions in the Seurat package (v3.1.2) [24] to perform an integrated analysis of multiple samples (accessed date: 28 Mary 2021). 

### 2.3. Human Gastric Adenocarcinoma Tissue Array and GC Specimens

Tissue microarray constructed using surgery resected gastric adenocarcinoma tumor/adjacent samples were purchased from Shanghai Outdo Biotech Company (HStmA180Su08, Accession date: 12, October, 2017). Immune-related adverse events can be irreversible and even fatal in patients with cancer and concomitant autoimmune disease [25,26]. Therefore, patients with autoimmune disease were excluded before immunotherapy in our study. Gastric cancer is a highly heterogeneous disease regarding the morphological and molecular characters compared with other cancer. In order to minimize the problems caused by heterogeneity in clinical diagnosis and treatment, multiple lesions were taken in clinical practice. For our tissue array samples, 180-dot sections were taken from the center of the tumor to the edge of the tumor during surgery. For the samples from patients before immunotherapy, 33% of samples were taken during surgery, the rest samples were taken by biopsy. Tissue sections for multiple fluorescence staining were collected before anti-PD-1/PD-L1 treatment. All the GC specimens were obtained from the tumor biobank of the Department of Gastrointestinal Oncology and Department of Pathology, Peking University Cancer Hospital and Institute. All providers granted the experimental applications with written informed consent. Clinical data were evaluated and recorded by clinicians and obtained from medical records. All procedures in this research observed the corresponding criteria of institutional ethics committee, Peking University Cancer Hospital with certificate no. 2019KT32. Mismatch repair (MMR) deficiency was determined by the lack of nuclear staining of one or more MMR proteins (MLH1, PMS2, MSH2, and MSH6) by immunohistochemistry. EBV-associated gastric carcinoma (EBVaGC) was determined by in situ hybridization (ISH) targeting EBV-encoded RNA (EBER) showing positive nuclei.

### 2.4. Multiplex Immunofluoresence Staining with Opal Fluorophores

Six fluorescence stainings were performed using a PerkinElmer Opal Kit (NEL811001KT, USA) with subsequent analysis by Vectra Automated Quantitative Pathology Imaging system and PerkinElmer InForm software2.4.0.rev0. The stained slides were evaluated blindly by two independent pathologists Dr. Xiaoyan Qiu and Dr. Jing Su. Five to ten fields in each specimen were selected randomly for analysis. Tissue segmentation algorithm was established by defining CK^+^ as tumor region and CK^-^ as mesenchymal region. CK and DAPI further identified membrane or cytosol protein expression pattern. The average intensity of the positively stained cells was given an intensity score from 0 to 3 (0 = no staining; 1 = weak; 2 = intermediate; 3 = strong staining). H-score was calculated as the following formula: H-score = [1 × (% of cell with weak intensity) + 2 × (% of cell with intermediate intensity) + 3 × (% of cell with strong intensity)] × 100. The final H-score ranging from 0 to 300 was automatically calculated by the PerkinElmer InForm software.

### 2.5. Bayes Model

Feature selection was based on the statistical analysis of the expression ratio of eight features (CMTM6, CMTM4, PD-L1, PD-1, CMTM6^+^PD-L1^+^, CMTM4^+^PD-L1^+^, CMTM4^+^6^+^, CMTM6^+^4^+^PD-L1 ^+^) on gastric tumor epithelial cells by MIS staining. One-way ANOVA analysis of eight features was done among the three groups (PD, PR, and SD) (Table S1). The CMTM4, CMTM6, and CMTM4^+^6^+^ that showed significant differences among the three clusters were included in the Bayes model. The naive Bayes model (NBM) was developed using the open source machine learning software Orange (https://orangedatamining.com/ (accessed on 12 October 2017)).

### 2.6. Statistical Analysis

ANOVA tests (multiple groups) with post-hoc Bonferroni correction was conducted when equal variance was assumed. Otherwise, Mann–Whitney U test or Kruskal–Wallis test was applied. Chi-squared test was used for association analysis of CMTM6 level and baseline characteristics (Table S2). Correlations were determined by Pearson r coefficient. Spearman correlation was computed if data are not in line with Gaussian distribution. Prognostic indicators (OS; PFS; estimated median survival time) were measured and formatted by the Kaplan–Meier method using SPSS 25.0. The Swimmer plot was constructed by Excel software and waterfall plots by SAS 9.4. Two-tailed *p* < 0.05 was considered to be of statistical significance.

## 3. Results

### 3.1. The Transcriptional Profile of CMTM6/4 and PD-L1 in Gastric Cancer Tissues

**Figure 1:**
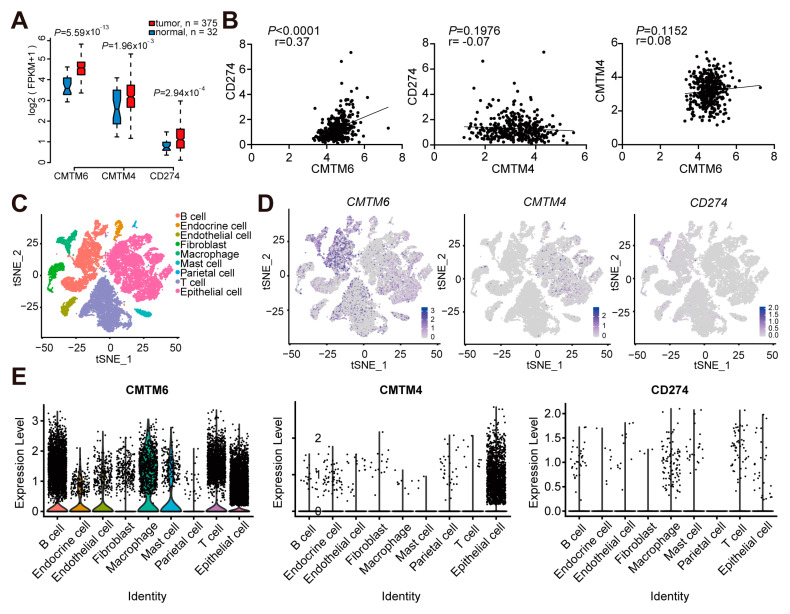
Cellular atlas of gastric tumors revealed different expression patterns of *CMTM6*, *CMTM4*, and *PD-L1* (Raspberry Pi). (**A**) Upregulated mRNA expression of *CMTM6*, *CMTM4*, and *CD274* in stomach adenocarcinoma (STAD) derived from TCGA. (**B**) Correlation of mRNA expression of *CMTM6*, *CMTM4*, and *CD274* by the TCGA dataset, analyzed by the Pearson test. (**C**) The t-SNE plot of high-quality single cells from gastric tumors and non-tumor gastric tissues to visualize cell-type clusters identified by the expression of known marker genes. (**D**) The t-SNE plots showing the expression levels of *CMTM6*, *CMTM4*, and *CD274* in the clusters described above. (**E**) Violin plots showing the smoothed expression distribution of *CMTM6*, *CMTM4*, and *CD274* in the clusters described above.

To study the expressional relationship of CMTM6/4 and PD-L1 in GC tissues, we first compared normal stomach tissues and the primary stomach adenocarcinomas (375 cases) obtained from The Cancer Genome Atlas (TCGA) database. As shown in Figure 1A, the tumor tissues had elevated levels of *CMTM6*, *CMTM4*, and *CD274 (PD-L1*) transcripts. The mRNA expression of *CMTM6* had a positive correlation with *CD274* in GC tissues (*p* < 0.0001, Figure 1B). No significant correlations were found between *CMTM4* and *CD274* (*p* = 0.1976), or between *CMTM4* and *CMTM6* (*p* = 0.1152).

To define the cell types in GC tissues with CMTM6/4 and PD-L1 co-expression, we further analyzed a recently published single-cell RNA sequencing (scRNA-Seq) dataset obtained from primary gastric adenocarcinoma [23]. This dataset comprises 27,677 high-quality single cells from nine tumor and three non-tumor samples. We orchestrated the cells into nine clusters by a series of population-specific genes expressed in different cell lineages (Figure 1C) [23]. t-SNE analysis showed that *CMTM6* mainly expressed in epithelial cells, T cells, B cells, and macrophages. *CMTM4* expression was specifically localized in epithelial cells. Although the level of *CD274* transcript was low, its expression on epithelial cells, T cells, and macrophages could be observed (Figure 1D,E). We thus focused on epithelial cells to investigate the relationship between CMTM6/4 and PD-L1 at the protein levels.

### 3.2. Membrane Co-Expression of CMTM6 and PD-L1 in Human Gastric Adenocarcinoma Tissues Array

**Figure 2:**
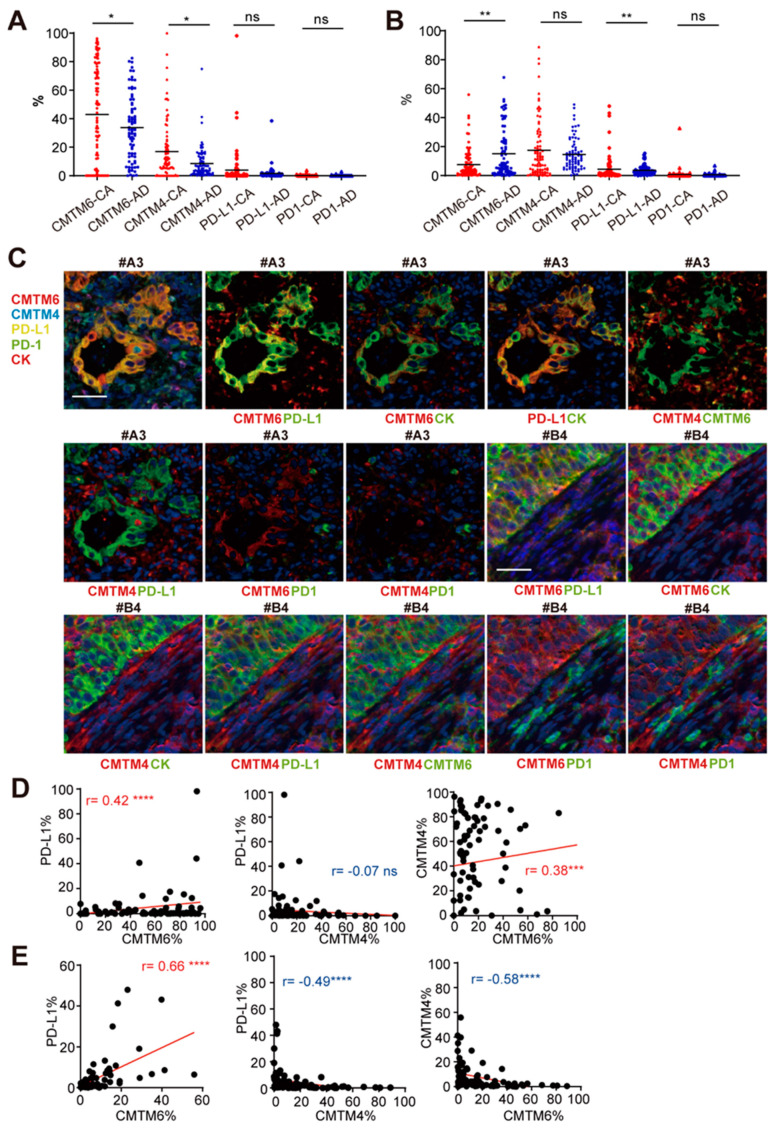
CMTM6 is elevated in tumor epithelial cells in gastric cancer tissues. (**A**,**B**) Expression of CMTM6, CMTM4, PD-L1, and PD-1 in the epithelial (**A**) and mesenchymal (**B**) region of 180-dot commercial gastric adenocarcinoma tissue microarray. CA: cancer, AD: Adjacent. (**C**) Representative images of co-localization pattern of indicated molecules in GC tissue microarray using multiplexed immunofluorescence staining (MIS), scale bar: 39μm. (**D**,**E**) Correlation analysis of CMTM6, PD-L1, and CMTM4 quantified by MIS in epithelial (**D**) and mesenchymal (**E**) region in the tissue microarray. * *p* < 0.05, ** *p* < 0.01, *** *p* < 0.001, **** *p* < 0.0001, “ns”, no significance, all by the Spearman test.

To study the protein expression profile and the relationship of CMTM6/4 and PD-L1 in patients with GC, we applied multiple immunofluorescence staining (MIS) by using multiplexed Opal fluorophores that can simultaneously evaluate six markers including CMTM6, CMTM4, PD-L1, PD-1, cytokeratin (CK), and DAPI (nuclear stain). The commercial tissue microarrays with 180 dots of gastric adenocarcinoma and adjacent normal tissues were first examined. The tumor tissues were divided into epithelial and mesenchymal regions by CK staining. In the epithelial region, the percentage of CMTM6 and CMTM4 single positive cells were significantly higher in the tumor tissues (*n* = 87) than the adjacent tissues (*n* = 78) (Figure 2A). The same results were found in the 69-paired samples within the 180-dot tissue array (*p* < 0.05 and *p* < 0.001, Supplementary Figure S1A). No significant difference in PD-L1 or PD-1 expression was observed between the tumor and adjacent epithelial tissues (Figure 2A). In the mesenchymal region, the frequency of PD-L1^+^ cells was higher in the tumor tissues while that of CMTM6^+^ cells was higher in the adjacent tissues (Figure 2B).

We next examined the co-expression profile of CMTM6/4 and PD-L1 in this 180-dot tissue array. CMTM6 expression was found in 80% of the tumor tissues while the co-localization of CMTM6 with PD-L1 on the surface of the tumor epithelial cells (CK^+^) was found in 28.74% of the tumor tissues (Figure 2C and data not shown). Among the CMTM4^+^ patients (75.86%), the co-localization of CMTM4 and PD-L1 varied and 52.73% of them showed CMTM4 staining in the epithelial as well as mesenchymal regions. The co-localization of CMTM4 with PD-L1 could be observed (Figure 2C, case #B4). The rest of the CMTM4^+^ samples showed the localization of CMTM4 only in the mesenchymal region with no PD-L1 expression (Figure 2C, case #A3). Neither epithelial nor mesenchymal regions in the 180-dot tissue array showed a significant co-localization of CMTM6/4 with PD-1 (Figure 2C).

We further used Spearman correlation analysis to examine the relationship between CMTM6/4 and PD-L1. In the epithelial region, CMTM6 expression showed positive correlation with PD-L1 (*p* < 0.0001, r = 0.42) and CMTM4 (*p* < 0.001, r = 0.38) (Figure 2D). In the mesenchymal region, CMTM6 expression had positive correlation with PD-L1 (*p* < 0.0001, r = 0.66), but negative correlation with CMTM4 (*p* < 0.0001, r = −0.58). A negative correlation of CMTM4 and PD-L1 was also found in the mesenchymal region (*p* < 0.0001, r = −0.49) (Figure 2E).

### 3.3. Higher Levels of CMTM6/4 Correlate with Poor Prognosis in GC Patients

**Figure 3:**
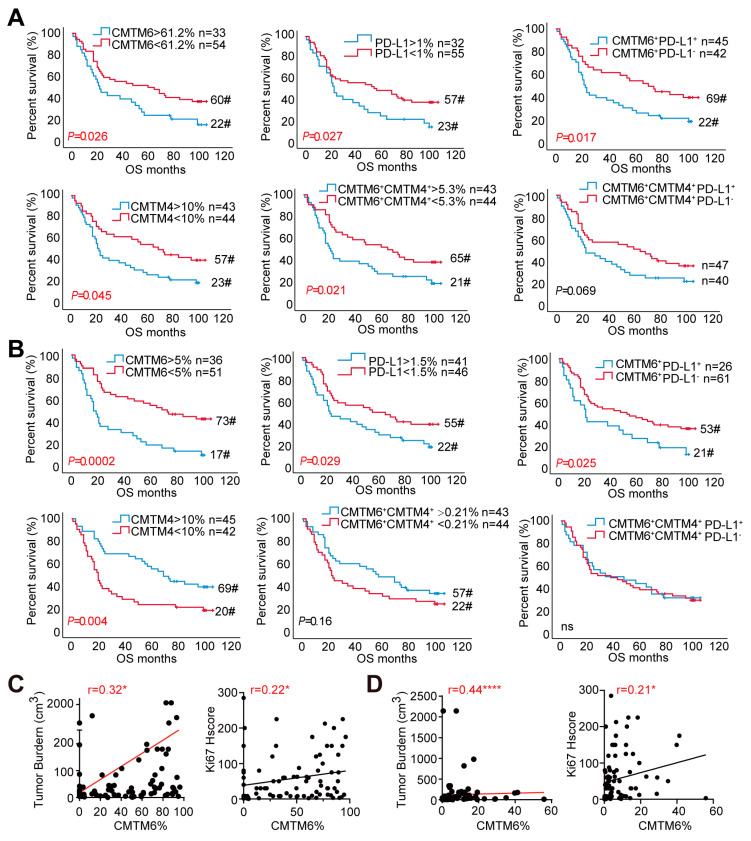
CMTM6 is associated with poor prognosis in the gastric adenocarcinoma. (**A**,**B**) Overall survival analysis of the above-mentioned tissue microarray samples by log rank test based on the expression of indicated molecules in the epithelial (**A**) and mesenchymal (**B**) regions. #, estimated median overall survival (months). (**C**,**D**) Correlation of CMTM6 and tumor burden as well as Ki-67 level in both epithelial (**C**) and mesenchymal (**D**) regions was calculated by the Spearman test. * *p* < 0.05, **** *p* < 0.0001.

We next investigated the prognostic value of CMTM6/4 and PD-L1 expression using the data derived from the 180-dot tissue array. In the tumor epithelial regions, the patients with higher percentages of CMTM6^+^, PD-L1^+^, or CMTM6^+^PD-L1^+^ cells had significantly shorter overall survival (OS) (*p* = 0.026; *p* = 0.027; *p* = 0.017, respectively). Similarly, the patients with higher percentages of CMTM4^+^ or CMTM4^+^CDTM6^+^ epithelial cells had significantly shorter OS (*p* = 0.045; *p* = 0.021, respectively) (Figure 3A). In the mesenchymal region, the patients with higher percentages of CMTM6^+^, PD-L1^+^, and CMTM6^+^PD-L1^+^ had significantly shorter OS (Figure 3B), while a high level of CMTM4 was associated with better OS (*p* = 0.004; median survival, 69 months vs. 20 months). Through multivariate analysis, CMTM6 was further shown as an independent risk factor in both epithelial (hazard ratio (HR), 1.761; 95% CI 1.058–2.931; *p* = 0.03) and mesenchymal regions (*p* = 0.0001) (Supplementary Figure S1B,C).

We further examined the association of CMTM6/4 expression with the tumor burden and found that high levels of CMTM6 in the epithelial (Figure 3C) and mesenchymal (Figure 3D) regions were positively associated with the tumor burden and cell proliferation (Ki-67). Such correlations were not found when CMTM4 was analyzed (Supplementary Figure S1D,E). We also did not find the association of CMTM6 or CMTM4 expression with clinical and pathological grade (Supplementary Figure S1F,G). Collectively, the tissue array data demonstrated that CMTM6 expression is associated with poor prognosis irrespective of epithelial or mesenchymal regions, while the association of CMTM4 and prognosis is more restricted to tumor epithelial region. Compared to each molecule alone, the co-expression of CMTM6 and PD-L1, CMTM6 and CMTM4 in the tumor epithelial region showed better prognostic value in the patients with GC.

#### 3.3.1. Co-Expression of CMTM6/4 and PD-L1 Is Associated with Better Response to Anti-PD-1/L1 Therapy in Gastric Adenocarcinoma Patients

**Figure 4:**
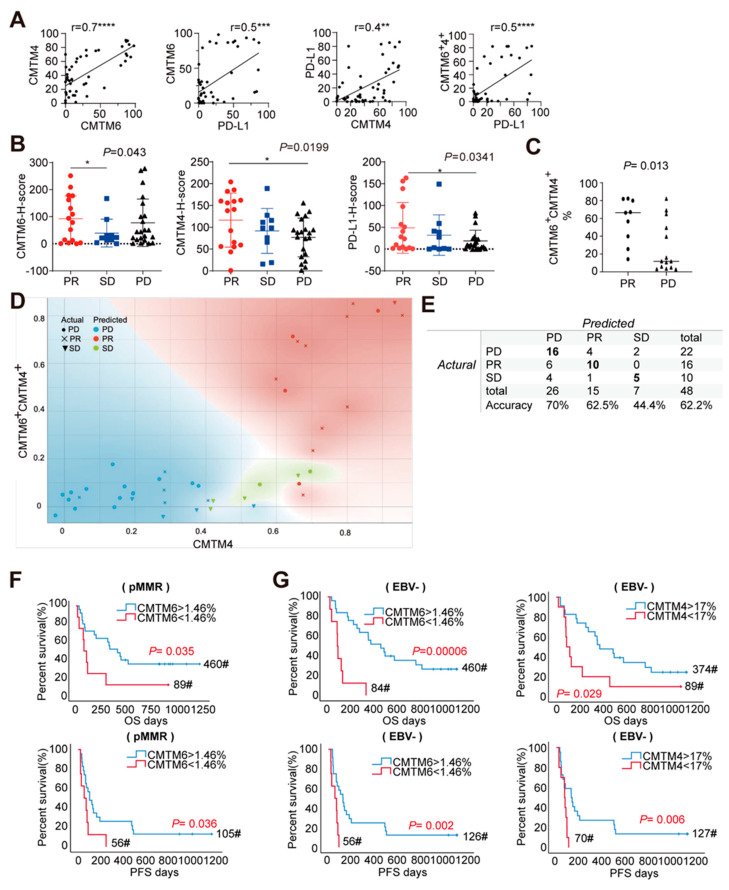
The expression of CMTM6 and CMTM4 is associated with short−term efficacy in anti−PD1/L1 treated patients. (**A**) Correlation analysis of epithelial CMTM6, CMTM4, and PD−L1 in the above-mentioned 48−case cohort by Spearman test. * *p* < 0.05, ** *p* < 0.01, *** *p* < 0.001, **** *p* < 0.0001. # estimated median overall survival (months). (**B**) Scatter plots showing CMTM6, CMTM4, and PD−L1 expression (H−score) in partial response (PR), stable disease (SD), or progressive disease (PD) group. (**C**) Scatter plots showing higher ratio of CMTM6^+^CMTM4^+^ in the PR group than PD group (CMTM6^+^CMTM4^+^% cutoff = 1%). (**D**) The naive Bayes model (NBM) was developed using the open source machine learning software Orange to classify of three groups based on the ratio of CMTM4 and CMTM6^+^4^+^. (**E**) The table indicated the predicted and actual accuracy of Bayes Model. (**F**) OS and PFS of epithelial CMTM6 expression in pMMR subpopulation (*N* = 35). (**G**) OS and PFS of epithelial CMTM6 or CMTM4 expression in EBV negative subpopulation (*N* = 35).

We next investigated whether the co-expression of CMTM6/4 and PD-L1 in GC is associated with the response to immunotherapy. A cohort of 48 GC patients receiving anti-PD-1/L1 treatment was analyzed (Supplementary Table S2 and Figure S2A–C). All the cases were classified as Stage IV by the pathological analysis. About 15 out of 48 cases (31.25%) achieved partial response (PR) in our cohort (Table S3). FFPE tumor tissue sections were collected before immunotherapy and were subjected to 6-color immunohistochemistry staining as described in the tissue array. The expression of CMTM6, PD-L1, and CMTM4 were examined and are illustrated in a matrix graph in Supplementary Figure S2A. As shown in Figure 4A, positive correlations were found between CMTM6 and PD-L1, CMTM4 and PD-L1, or CMTM6 and CMTM4 expressions in the epithelial region (r = 0.5; r = 0.4, r = 0.7), being largely consistent with the tissue array data. A strong association was also found between PD-L1 and CMTM6^+^CMTM4^+^ expression (Figure 4A, r = 0.5, *p* < 0.0001). In addition, the patients with two or more metastatic sites had relatively higher CMTM6 level (*p* < 0.05) than those with only one metastatic lesion (Table S2). 

To examine whether the expressions of these molecules are associated with patient response to PD-1/L1 therapy, the GC patients were divided into different response groups (partial response (PR), stable disease (SD), and progressive disease (PD)) and the histochemistry (H)-score of each molecule was compared in the epithelial region. As shown in Figure 4B, the patients in PR group showed higher expression of CMTM6, CMTM4, and PD-L1 when compared to those in either the SD or PD groups. When CMTM6 and CMTM4 double positive cells were calculated, a larger difference was found between PR and PD groups (Figure 4C). We further used the Bayes model to examine whether the expression of these molecules could be used to predict the immunotherapeutic outcome. As shown in Figure 4D,E, the frequencies of CMTM6^+^4^+^ and CMTM4^+^ cells in the tumor epithelial region had better predictive probability, with the total predictive accuracy being 62.2% and the accuracy of PD, PR, and SD being 70%, 62.5%, and 44.4%, respectively. These results indicate that higher total protein levels of CMTM6 and CMTM4 in the tumor epithelial region are associated with better short-term efficacy of immunotherapy. 

We also examined whether CMTM6/4 level could indicate a difference in the long-term after immunotherapy. In this small cohort, the OS and PFS were not significantly different between the patients with high and low expression of CMTM6, CMTM4, and PD-L1 (Supplementary Figure S2D,E). When the patients with pMMR were analyzed, high expression of CMTM6 was associated with longer OS as well as PFS. When EBV positive patients were excluded, longer OS and PFS were also found in the patients with high expression of CMTM6 or CMTM4 (Figure 4F,G). However, no significance was reached when the co-expressions of CMTM6 and PD-L1, CMTM4 and PD-L1 were analyzed. These results indicate that in the patients receiving PD-1/L1 immunotherapy, CMTM6/4 expression had better long-term predictive value in those pMMR or EBV negative patients.

#### 3.3.2. Co-Expression of Membrane CMTM6/4 and PD-L1 Is Beneficial to Precision Patients Screening

**Figure 5:**
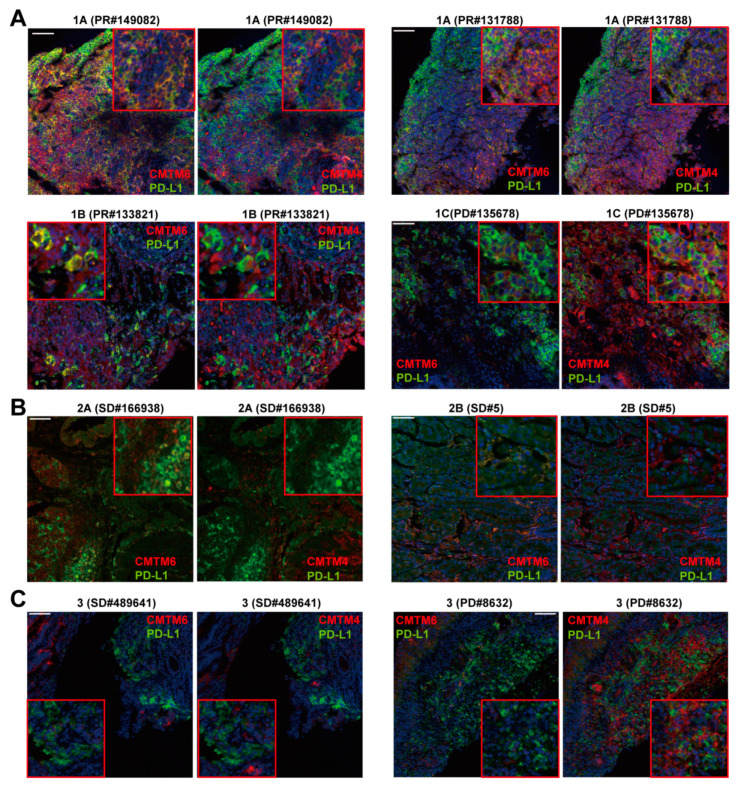
The pathological patterns of CMTMs and PD-L1 predict the efficacy of immunotherapy. (**A**) Pattern 1A was identified as CMTM6^+^CMTM4^+^PD-L1^+^ on the membrane of tumor epithelium, as shown in representative figures of case #149082. Pattern 1B showed the co-expression of CMTM6 and PD-L1 on the membrane of tumor epithelial cells without CMTM4 expression. Pattern 1C showed the co-expression of CMTM4 and PD-L1 on the membrane of tumor epithelial cells without CMTM6 expression. (**B**) Pattern 2A showed the co-expression of CMTMs with PD-L1 in the mesenchymal cells. Pattern 2B showed the co-expression of CMTM4 and CMTM6 without PD-L1 in the epithelial cells. (**C**) Pattern 3 showed single positive of CMTM6, CMTM4, and PD-L1 in different cells. Scale bar: 100μm.

We further investigated whether the expression pattern of CMTM6/4, instead of the percentage of positive cells or H-score, could be used to select patients for PD-1/L1 therapy. Three different expression patterns were identified by two pathologists. Pattern 1 was identified as CMTM6 and/or CMTM4 co-localized with PD-L1 on the membrane of tumor epithelial cells (CK^+^). The majority of patients with pattern 1 (91.66%) reached PR or SD after anti-PD-1/L1 immunotherapy. Pattern 1 could be further divided into three subsets. CMTM6^+^CMTM4^+^PD-L1^+^ and CMTM6^+^PD-L1^+^ on the membrane of tumor epithelium were identified as patterns 1A and 1B, respectively (Figure 5A, Supplementary Table S4). All six patients that fell in patterns 1A and 1B reached PR in response to the immunotherapy (Supplementary Table S4). Pattern 1C had CMTM4^+^PD-L1^+^ on the membrane of tumor epithelium. The six patients that fell in pattern 1C had different responses to the immunotherapy (PR (3/6), SD (2/6), and PD (1/6)) (Figure 5A, Supplementary Table S4). 

Pattern 2A had co-expression of CMTM6/4 and PD-L1 in the mesenchymal cells. More than half of the patients with pattern 2A were PD (2 PR, 2 SD, and 6 PD) (Figure 5B). The tumor epithelium with CMTM6^+^CMTM4^+^ but PD-L1^−^ in the same cells was classified as pattern 2B. Unlike pattern 1, the intensity of CMTM6 and CMTM4 expression in pattern 2B was lower and was mainly found in the cytoplasm of tumor epithelial cells (Figure 5B, Supplementary Table S3). The samples with single positive CTMT6, CTMT4, or PD-L1 expression in tumor epithelial or mesenchymal cells were classified as pattern 3. No co-localization of CMTM6 or CMTM4 with PD-L1 was detected. The majority of the patients (83.33%) with pattern 3 did not reach PR (PD 66.67%; SD 16.67%) (Figure 5C, Supplementary Table S4). Collectively, these data demonstrated that pattern 1 with membrane expression of CMTM6^+^PD-L1^+^ and/or CMTM4^+^PD-L1^+^ on the surface of tumor epithelium is associated with better response to anti-PD-1/L1 therapy. The membrane co-expression of CMTM6/4 with PD-L1 on the tumor epithelial cells may thus be a promising criterium for precision therapeutic patient stratification.

## 4. Discussion

PD-L1 is one of the primary biomarkers in the stratification of patients receiving anti-PD-1/L1 therapy [1,6,7]. In some of the clinical trials with GC, however, high PD-L1 expression alone did not correlate well with the response rate of anti-PD-1/L1 therapy [2,5,9,10]. By developing a multiplex immunofluorescence panel with antibodies against CK, CMTM6, CMTM4, PD-L1, PD1, and DAPI, we demonstrated that the co-localization of CMTM6/4 with PD-L1 on the membrane of GC epithelium had better prognostic value than PD-L1 alone, and better predictive value of short-term efficacy of anti-PD-1/L1 therapy.

In the scRNA-Seq data from primary gastric adenocarcinoma [23], CMTM6 showed high expression on epithelial cells, T cells, B cells, and macrophages, while CMTM4 was specifically localized on epithelial cells. The multiplexed immunofluorescence staining also confirmed that CMTM6 or CMTM4 could co-localize with PD-L1 on the same epithelial cells. This is consistent with CMTM6/4 mRNA expression on epithelium. A recent study indicates that CMTM6 is expressed on CD8^+^ T cells and CD163^+^ M2 macrophages in colorectal cancer tissues. Its expression in T cells and M2 macrophage is associated with clinical benefit and response to immunotherapy [27]. Another study found the expression of CMTM6 mainly on CD68 positive macrophages in the non-small-cell lung cancer (NSCLC), and its expression is correlated with longer overall survival in the patients with immunotherapy [28]. We also found CMTM6/4 and PD-L1 expression in the mesenchymal region, suggesting that the positive cells might be macrophages or T cells. Thus, the cell type specificity and the role of CMTM6/4 in these cells in the gastric tumor microenvironment need further investigation.

The levels of CMTM6 mRNA and protein were both applied for the prognosis analysis of different types of tumor and contradictory conclusions were obtained. At the mRNA level, a high level of CMTM6 is correlated with better survival in gastric cancer, colorectal cancer [20,29], while high level of CMTM6 is correlated with worse survival in gliomas [16]. At the protein level evaluated by immunohistochemistry, highly expressed CMTM6 indicates poor prognosis in gastric cancer, HNSCC, hepatocellular carcinoma [21,30,31,32]. In our 180-dot tissue array with gastric adenocarcinoma, we confirmed that a high protein level of CMTM6 was correlated with worse survival and the co-expression of CMTM6 enhanced the prognostic value of PD-L1 in gastric cancer. Importantly, the expressions of both epithelial and mesenchymal CMTM6 had prognostic values. The expression of CMTM4 was different and the high level of epithelial CMTM4 was associated with worse survival while that of mesenchymal CMTM4 with better survival. The co-expression of CMTM6 and CMTM4 enhanced the prognostic value of epithelial CMTM4 in gastric cancer. Together, our data highlight the difference of CMTM6 and CMTM4 in the prognosis of gastric cancer. 

Our results further indicate three common features inCMTM6 expression in gastric cancer as well as other types of tumor. This includes elevated expression in tumor tissues, correlation with worse prognosis, and association with PD-L1 expression. Additionally, CMTM6 has three special traits in gastric adenocarcinoma: (1) CMTM6 localizes not only on the membrane of tumor epithelium, but also in cells in the mesenchymal region; (2) CMTM6 protein expression level is positively correlated with tumor burden or more metastatic sites; and (3) CMTM6 co-expresses with CMTM4 on the gastric tumor epithelial cells and mesenchymal cells. These special traits provide valuable indication for anti-PD1/L1 therapy. Indeed, our results demonstrate that the co-expression of CMTM6 or CMTM4 with PD-L1 on the membrane of tumor cells is critical for immunotherapeutic response and the pathological patterns of their expression may provide detailed information to conduct personalized immunotherapy. 

PD-L1 expression in tumor cells is regulated at both mRNA and protein levels. On one hand, the tumor intrinsic mechanisms including genetic and epigenetic alterations, oncogenic and tumor suppressor signals are linked to PD-L1 mRNA expression. PI3K-AKT-mTOR and RAS-MAPK-p38/ERK are two pivotal pathways involved in this process. On the other hand, PD-L1 protein is modulated by phosphorylation, glycosylation, palmitoylation, and poly-ubiquitination. The phosphorylation of S195 (by p-AMPK), T180, or S184 (by GSK3β) of PD-L1 mediates its degradation, while Sigmal 1, FKBP51s, and B3GNT3 maintain its protein stability by N-glycosylation [19]. Recent studies indicate that high expression of PKM2 synergizes with PD-L1 to predict worse survival in human lung adenocarcinoma, while WSX1 downregulation is closely correlated with poor prognosis in HCC. WSX1 activates GSK3β-mediated PD-L1 degradation by transcriptionally suppressing PI3Kδ/AKT signal [33]. However, whether WSX1 or PKM2 could be an indicator for the response to anti-PD-1/L1 therapy, and whether CMTMs have interaction or synergy effect with these signaling pathways are awaited for further investigation.

## 5. Conclusions

In summary, the co-expression of CMTM6/4 with PD-L1 on the membrane of gastric cancer cells predicts poor prognosis as well as better short-term efficacy of immunotherapy with anti-PD-1/L1 antibodies. The ability of maintaining membrane expression of PD-L1 endows CMTM6/4 with a pivotal role in precise patient stratification in the clinical settings.

## Data Availability

Datasets of stomach cancer (STAD) from The Cancer Genome Atlas (TCGA) were downloaded from UCSC Xena web browser (https://xenabrowser.net/ (accessed on 28 October 2020)). Gene expression datasets of human cancer cell lines were directly downloaded from CCLE (https://portals.broadinstitute.org/ccle (accessed on 28 October 2020)). Single-cell datasets of raw UMI count matrices were derived from a recent study with accession number HRA000051 (http://bigd.big.ac.cn/gsa-human/ (accessed on 28 March 2021)).

## Supplementary Materials

The following are available online at https://www.mdpi.com/article/10.3390/cancers13205175/s1, Figure S1: The expression of CMTM6 and CMTM4 in 180-dots tissue array, Figure S2: The expression of CMTM6, CMTM4, and PD-L1 in 48 GC patients before receiving immunotherapy, Table S1: One-way ANOVA analysis of 8 features in tumor epithelial cells in the 48-case with immunotherapy, Table S2: Baseline Characteristics of the 48 GC patients receiving immunotherapy, Table S3: Efficacy outcomes in evaluable 48-case patients, Table S4: Expression patterns of CMTMs and PD-L1 in the 48-case gastric cancer tissues.
