# Supplementary material for: Co-Expression with Membrane CMTM6/4 on Tumor Epithelium Enhances the Prediction Value of PD-L1 on Anti-PD-1/L1 Therapeutic Efficacy in Gastric Adenocarcinoma
Source: Cancers (Basel). 2021 Oct 15;13(20):5175. doi: 10.3390/cancers13205175 (PMC8533876; doi:10.3390/cancers13205175)
Supplement: Supplementary file 1 [file cancers-13-05175-s001.zip › 20210928-supplementary figures.pdf]

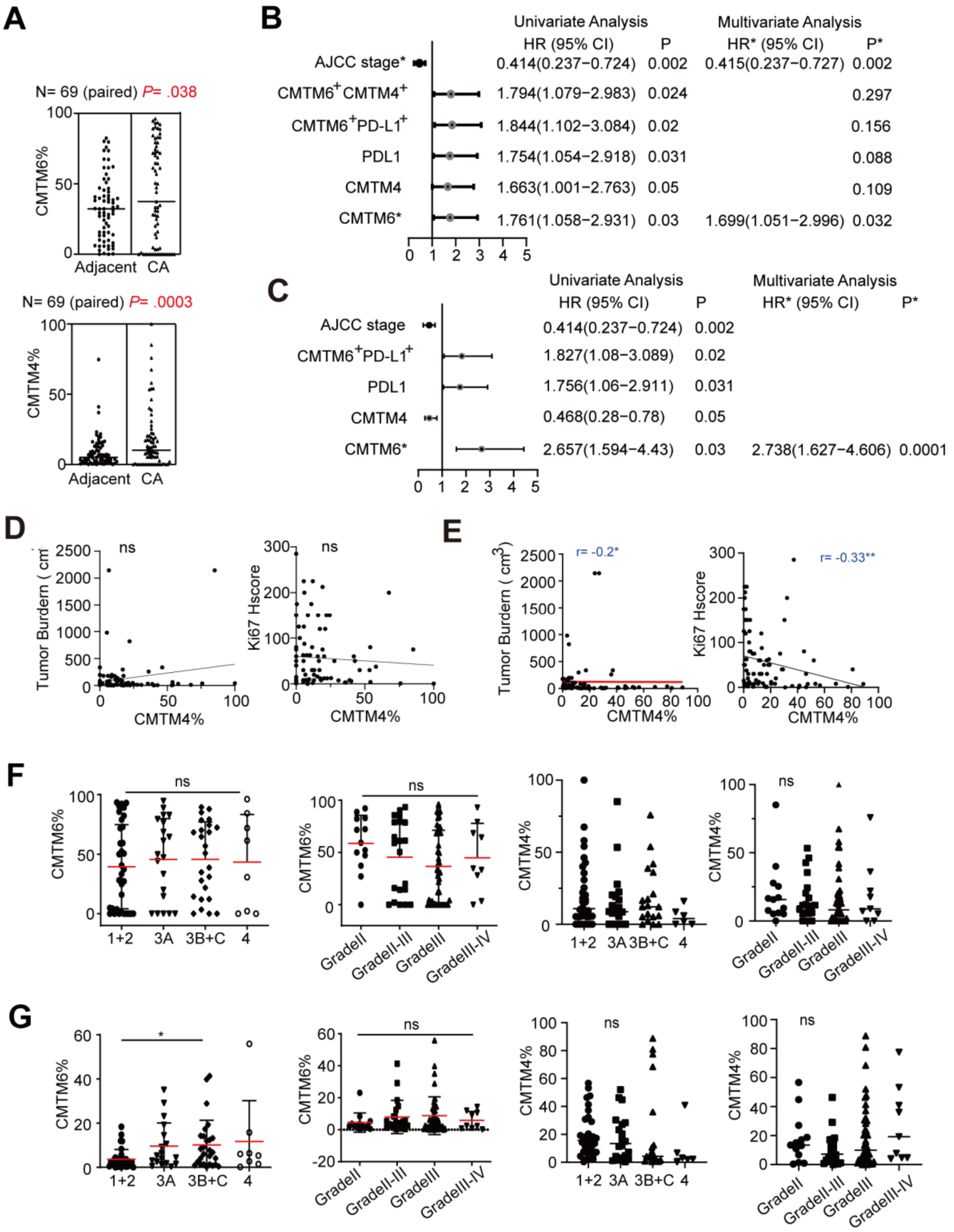

Supplementary Figure S1. The expression of CMTM6 and CMTM4 in 180-dots tissue array. A. Comparison of epithelial CMTM6 and CMTM4 expression in 69-paired GC and the adjacent tissue in the TMA. B-C. Forest plot of hazard ratios and 95% CIs for all-cause mortality in both epithelial (B) and mesenchymal (C) region by Cox-hazard model. D-E. Correlation of CMTM4 and tumor burden as well as Ki-67 level in both epithelial (D) and mesenchymal (E) region was calculated by Spearman test. *, $p < 0.05$, **, $p < 0.01$. F. The level of epithelial CMTM6 in different clinical stages as well as pathological stages was analyzed by one-way ANOVA tests. G. The level of epithelial CMTM4 in

different clinical stages as well as pathological stages was analyzed by one-way ANOVA tests.

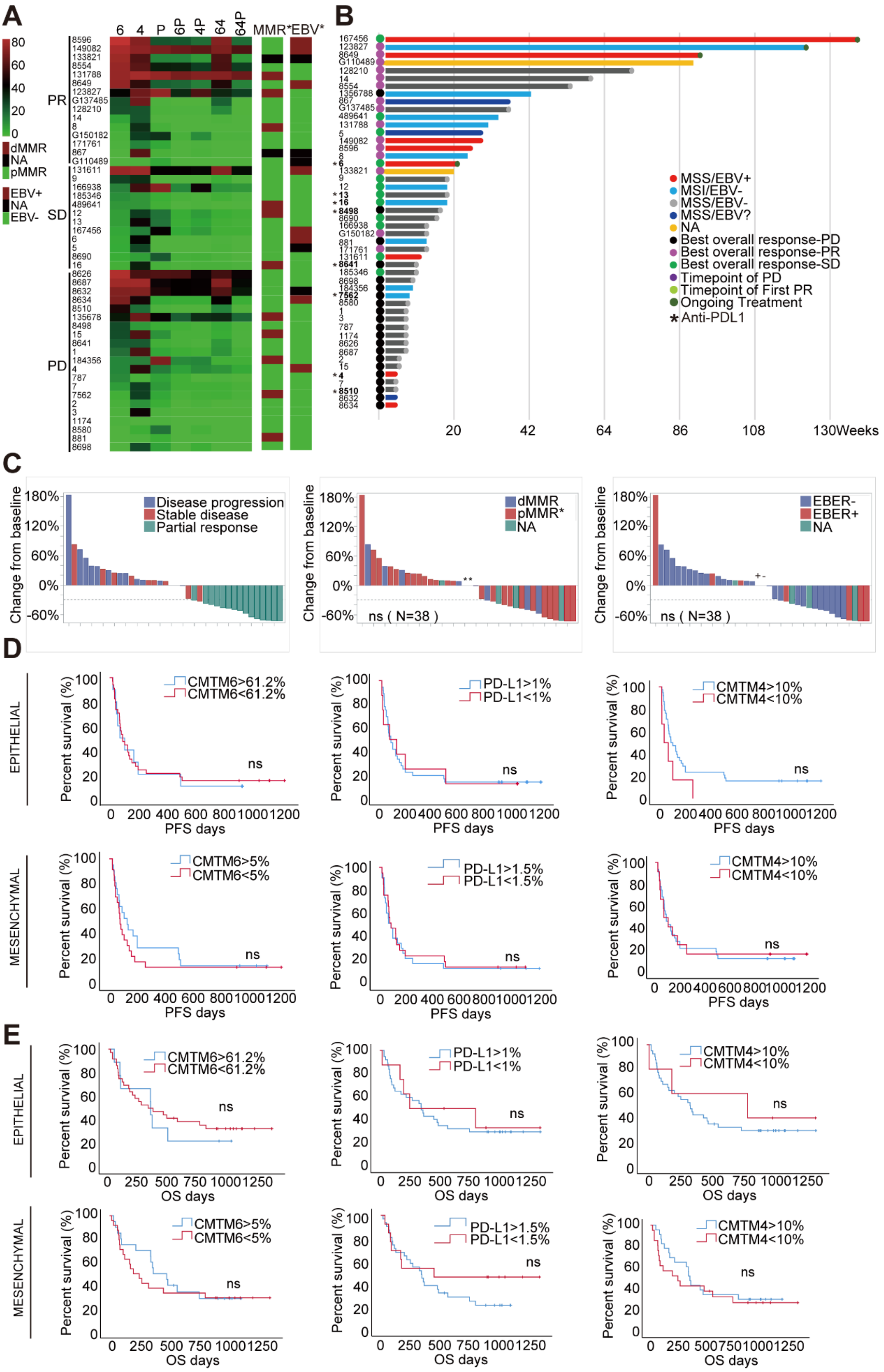

Supplementary Figure S2. The expression of CMTM6, CMTM4, and PD-L1 in 48 GC patients before receiving

immunotherapy. A. Integrated expression signature quantified by multiple immunofluorescence staining of GC patients. Mismatch repair status (pMMR/dMMR, proficient or deficient mismatch repair) and EBV positivity were included. (6: $CMTM6^+$, 4: $CMTM4^+$, P: PD-$L1^+$,6P: $CMTM6^+$ PD-$L1^+$, 4P: $CMTM4^+$ PD-$L1^+$, 64: $CMTM6^+CMTM4^+$, 64P: $CMTM6^+CMTM4^+$ PD-$L1^+$). B. Swimmer's plot illustrates the major medical events of each patient. The length of each lane represents the duration of immunotherapy. NA, EBV and MMR status not available. C. Waterfall plots with known immune related biomarkers as grouping factors of 38 patients receiving immunotherapy with tumor burden change data. According to RECIST1.1, Y-axis represents sum of diameters of target lesions compared with baseline respectively. D. Progress free survival (PFS) of tumor epithelial $CMTM6^{hi}$ versus $CMTM6^{lo}$ (the same cutoff as indicated in Figure 5A-B), PD-$L1^{hi}$ versus PD-$L1^{lo}$, $CMTM4^{hi}$ versus $CMTM4^{lo}$ in the 48-case cohort after immunotherapy. E. Overall survival (OS) of tumor epithelial $CMTM6^{hi}$ versus $CMTM6^{lo}$ (the same cutoff as indicated in Figure 5A-B), PD-$L1^{hi}$ versus PD-$L1^{lo}$, $CMTM4^{hi}$ versus $CMTM4^{lo}$ in the 48-case cohort after immunotherapy.
