# Supplementary material for: Co-Expression with Membrane CMTM6/4 on Tumor Epithelium Enhances the Prediction Value of PD-L1 on Anti-PD-1/L1 Therapeutic Efficacy in Gastric Adenocarcinoma
Source: Cancers (Basel). 2021 Oct 15;13(20):5175. doi: 10.3390/cancers13205175 (PMC8533876; doi:10.3390/cancers13205175)
Supplement: Supplementary file 1 [file cancers-13-05175-s001.zip › supplementary Tables.pdf]

Table S1. One-way ANOVA analysis of 8 features in tumor epithelial cells in the 48-case with immunotherapy.

Feature	CMTM6	CMTM4	PD-L1	PD-1	6^+PD-L1$^+$	4^+PD-L1$^+$	4^+6^+	6^+4^+PD-L1$^+$
significance	0.155	0.02	0.54	0.248	0.185	0.159	0.058	0.162

Table S2 Baseline Characteristics of the 48 GC patients receiving immunotherapy

	Total(N=48)	CMTM6		*p*
		Higher(>Median*)	Lower(<Median*)	
Age (yr)	61 (24-81)			0.56
>=60	28	15	13	
<60	20	9	11	
Sex				0.75
Female	13	7	6	
Male	35	17	18	
ECOG status				0.35
0	20	12	8	
1	24	11	13	
2	4	1	3	
Previous gastrectomy				0.087
Yes	21	8	13	
No	27	17	10	
Treatment naive	3			
Number of previous therapy(palliation)				0.5
1	18	8	10	

2	15	9	5	
>2	12	7	5	
Primary tumor site				0.26
Cardia/Fundus/EGJ	21	10	11	
Body	11	8	3	
Antrum	14	7	7	
GSC**	2	0	2	
Metastatic sites				0.046
1	12	3	9	
>=2	36	21	15	
EBV positivity	18.6% (8/43)			0.14
EBER+	8	6	2	
EBER-	35	16	19	
HER2 positivity	48.9%（23/47）			0.46
+	23	13	10	
- (FISH-/IHC-)	24	11	13	
pMMR	76.1% (35/46)	18	17	0.73
dMMR	23.9% (11/46)	5	6	

*, Median level, 14.8%. **, GSC, gastric stump carcinoma. Treatment naïve, first diagnose without chemo or surgery

Table S3 Efficacy outcomes in evaluable 48-case patients

	Total(N=48)
Best overall response	
Complete response	0
Partial response	15
Stable disease	12
Progressive disease	21
Time to response (weeks)	8 (6-36)
Duration of response (weeks)	34 (8-78)
Median progression-free survival (95%CI; weeks)	15.0 (8.8-21.2)
Median overall survival (95%CI; weeks)	50.1 (33.9-66.3)

Table S4. Expression patterns of CMTMs and PD-L1 in the gastric cancer tissues

MSI Expression pattern	Tumor epithelial membrane	mesenchymal	Patients	Short-term efficacy of ICB
Pattern 1<br>Tumor<br>CMTM6/4^{+}PD-L1^{+}	**1A:**CMTM6^{+}CMTM4^{+}PD-L1^{+}		**PR149082**, PR8554, PR131788,	PR(9/12) 75%<br>SD(2/12) 16.66%<br>PD(1/12) 8.33%<br>Non-PD (11/12) 91.66%
	**1B:**CMTM6^{+}CMTM4^{-}PD-L1^{+}		PR8,**PR133821**, PR8596	
	**1C:**CMTM6^{-}CMTM4^{+}PD-L1^{+}		**PD135678**,PRG180182, SD131611, SD8690, PR123827, PR8649,	
Pattern 2<br>Mesenchymal<br>CMTM6/4^{+}PD-L1^{+}<br>OR Tumor<br>CMTM4^{+}6^{+} PD-L1^{-}		**2A:**CMTM6^{-}CMTM4^{+}PD-L1^{+}or CMTM6^{+}CMTM4^{-}PD-L1^{+}	PR171761, PR128210, **SD166938**, SD185346, PD8580,PD1,PD4, PD7562, PD8498,PD184356,	PR(3/18) 16.67%<br>SD(7/18) 38.89%<br>PD(8/18) 44.44%
	**2B:**CMTM6$^{+/lo}$CMTM4$^{+/lo}$PD-L1^{-}		PR14,PD8641, PD8626, SD13, **SD5**, SD6, SD9, SD16,	
Pattern 3<br>Single positive	**3A:**<br>CMTM6^{+}<br>or CMTM4^{+}<br>or PD-L1^{+}	**3A:** with or without<br>CMTM6^{+}<br>or CMTM4^{+}<br>or PD-L1^{+}	PRG137485, PR86710, PRG110489, **SD489641**, SD12 , SD167456, **PD8632**, PD8698, PD3, PD1174, PD8510, PD7, PD15, PD8687, PD787, PD2, PD881, PD8634	PR(3/18) 16.67%<br>SD(3/18) 16.67%<br>PD(12/18) 66.67%<br>Non-PR (15/18) 83.33%
